# Inflammatory Pathology and Mechanisms of Filamentous Fungal Infection in Breast Implants: A Commentary

**DOI:** 10.3390/pathogens15040362

**Published:** 2026-03-29

**Authors:** Andrew W. Campbell, Abdelrahman Elamin

**Affiliations:** 1 MyMycoLab, Land O Lakes, FL 34639, USA; 2 Faculty of Science and Engineering, Teikyo University, Utsunomiya 320-8551, Tochigi, Japan; elamin_555@hotmail.com

**Keywords:** breast implant, fungi, infection, biofilm, inflammatory signs, mycotoxins, mechanism, systemic symptoms

## Abstract

Filamentous fungal infections of breast implants remain underrecognized compared with infections caused by Candida species and bacteria, despite their potential to induce significant inflammatory pathology. This commentary highlights the distinctive ability of filamentous fungal biofilms to penetrate deeply into the implant capsule, a feature that may contribute to distinct pathogenic behaviour compared with bacterial or yeast biofilms. We discuss how this invasive behavior may contribute to the perception that reported cases represent the first documented breast implant-associated fungal infections, when in fact such infections may be under-recognised and under-investigated. By drawing attention to these mechanisms and their clinical implications, this commentary aims to stimulate greater awareness and further investigation within the fields of infectious diseases and pathogen research.

Breast implant surgery is increasingly recognized as being associated with a range of complications. The most frequently reported issues include capsular contracture, implant rupture, seroma formation, and infection, and longer follow-up periods reveal a greater incidence of late-onset complications, underscoring the importance of extended monitoring after implantation [1]. Although these concerns have gained renewed attention in recent years, they were first highlighted decades ago by the U.S. Food and Drug Administration (FDA), which, in 1992 [2], restricted the use of silicone gel-filled implants to clinical trials due to insufficient long-term safety data. Subsequent FDA communications—including strengthened safety requirements in 2021 [3] and safety updates in 2022 and 2023 [4] reporting cases of squamous cell carcinoma (SCC) arising in the implant capsule—reflect ongoing uncertainty regarding the full spectrum of implant-related risks. Despite these repeated regulatory actions, many of the original concerns remain unresolved, continuing to pose challenges for patient safety and oversight. Importantly, the emergence of late-onset complications such as SCC and chronic inflammatory presentations highlights the need for systematic evaluation of overlooked infectious aetiologies, including filamentous fungi, as improved microbiological characterisation may meaningfully inform both clinical management and regulatory responses. This article presents a narrative commentary that integrates selected case observations with emerging experimental findings to explore the potential role of filamentous fungal infections in producing inflammatory signs and associated symptoms, with the aim of generating hypotheses and conceptual connections rather than providing a comprehensive systematic review.

Because implant-related symptoms can arise from multiple, distinct processes, breast implant illness (BII) refers to a constellation of systemic, patient-reported symptoms—including fatigue, cognitive difficulties, musculoskeletal pain, hair loss, anxiety, and autoimmune-like complaints—in individuals with breast implants. BII is a symptom-based, culture-negative construct and is distinct from the culture-proven filamentous fungal infections described in this report. However, it is possible that in a subset of patients, symptoms attributed broadly to BII may, in fact, reflect an unrecognized underlying infectious process, including filamentous fungal involvement. While several studies have noted symptom improvement after explantation [5,6], the underlying drivers remain uncertain and may reflect a direct pathological process, a perceived association with implant placement, or a combination of both [7].

Breast reconstruction and augmentation are frequently complicated by periprosthetic infection [8]. Most studies in the literature on breast implant infections emphasize bacterial pathogens or *Candida* [9,10,11]. Concentrating on filamentous fungi highlights a less explored yet clinically significant dimension. With respect to their penetration ability, Harding et al. (2009) [12] challenged the traditional view that biofilms are restricted to bacteria and yeasts, demonstrating that filamentous fungi are also capable of forming complex biofilm structures. In their analysis, they describe how fungal biofilms differ fundamentally from those of unicellular organisms: rather than aggregates of single cells embedded in extracellular polymeric substances, filamentous fungal biofilms consist of interconnected hyphal networks surrounded by extracellular matrix. This architecture provides both mechanical strength and invasive capacity. The authors emphasize that hyphal growth and specialized infection structures enable filamentous fungi to adhere tightly to surfaces and penetrate substrates—a mechanism distinct from the enzymatic degradation and pseudohyphal extension seen in yeasts or the extracellular polymer secretion typical of bacteria. These insights highlight the importance of considering filamentous fungi in discussions of implant infection and permeability. From the early 2000s to the present, several reports have described filamentous fungal infections of breast implants, typically noting that such infections are rare complications. Table 1 provides a summary of reported cases, which are examined in greater detail in the subsequent sections. In line with these observations, the available case literature suggests that filamentous fungal breast implant infection is a rare but potentially under-recognised complication.

Kuhn and Homsy (2022) [13] described a 39-year-old woman who developed unilateral breast swelling, tenderness, and erythema two decades after augmentation mammaplasty. In addition to local inflammatory signs, she reported systemic symptoms including fatigue, brain fog, unusual body odor, and difficulty losing weight. Examination revealed severe capsular contracture, and implant removal uncovered purulent drainage, a white coating, and turbid green fluid with debris. Cultures identified *Propionibacterium acnes* and an unspecified *Penicillium* species, marking the first documented fungal association with BII in a saline implant.

In the discussion presented in [13], the authors draw on prior references to emphasize that BII has traditionally been linked to bacterial biofilms, particularly *P. acnes*, which can trigger autoimmune-like syndromes described as “human adjuvant disease.” Although fungal infections are rare, usually involving *Candida* or *Aspergillus*, they noted that fungal antigens may provoke similar immune responses. Wright et al. (2006) [16], for example, proposed that implant membranes may be semipermeable to fungal spores, permitting contamination from surrounding tissue or blood. The simultaneous fungal growth inside and outside the intact implant strongly supports this mechanism.

This case raises the possibility that fungal involvement may be overlooked in some patients with implant-related symptom complexes. Roughly one quarter of breast infections yield negative cultures, and fungal cultures are not routinely performed. By documenting fungal presence alongside bacterial biofilm, Kuhn and Homsy expand the understanding of implant-related complications and situate fungal infection within the wider discussion of biofilm-mediated immune activation.

Koan et al. (2022) [14] reported the case of a woman who developed bilateral breast implant infection five years after augmentation mammaplasty. She presented with breast swelling, tenderness, and purulent fluid collections, consistent with local inflammatory signs. The implants were removed before cultures were obtained, and intraoperative histopathology demonstrated budding hyphae and right-angle branching. Cultures later identified *Scedosporium apiospermum* from the right breast and its teleomorph *Pseudallescheria boydii* from the left, representing the first documented breast implant-associated infection with this mold in an immunocompetent patient.

The discussion in [14] emphasizes that breast implant infections are most often bacterial, particularly *Staphylococcus aureus*, and are frequently linked to biofilm formation on implant surfaces. While fungal infections are rare, previous reports have described *Aspergillus*, *Candida*, and *Trichosporon*. This case expands the spectrum by demonstrating that molds such as *Scedosporium apiospermum* can also be implicated. The authors highlight that diagnosis is challenging because histopathology may not show distinctive features, making culture essential. By situating *Scedosporium* within the broader context of biofilm-associated implant infections, Koan et al. underscore the fact that filamentous fungi, though uncommon, should be considered in the differential diagnosis of late-onset breast implant complications.

Dinolfo (2013) [15] described an inflammatory response in the soft tissue surrounding a breast implant complicated by fungal infection. A 27-year-old woman developed a persistent postoperative infection of her left breast following bilateral implantation, presenting with erythema, induration, and purulent drainage unresponsive to antibiotics. Subsequent cultures identified *Aspergillus fumigatus*, and removal of the prosthesis revealed it was completely encased in mold. The author attributed the infection to colonization of the implant itself, most likely introduced during or after surgery. Notably, epidemiologic review found no evidence of additional cases or manufacturer defects, supporting the conclusion that this was an isolated contamination event.

Wright et al. (2006) [16], cited in [13] as providing evidence of the permeability of silicone implants to fungi, presented an important case report documenting fungal infection and referencing other related cases. The authors reported on a 31-year-old woman with a three-day history of pain and sudden enlargement of the right breast, occurring 18 months after cosmetic bilateral subpectoral augmentation with saline-filled implants. At surgical exploration, the intact right implant was surrounded by gelatinous yellow fluid and contained turbid material. Following removal, *A. flavus* was cultured from both the luminal and pericapsular fluid of the implant. Histopathological examination of the pericapsular tissue demonstrated a lymphocytic infiltrate and granuloma formation associated with branching fungal hyphae indicative of *Aspergillus*.

The authors described the mechanisms of fungal infection by referencing previous studies, which proposed several possible routes of colonization of breast implants. These include airborne dissemination, contamination during manufacture, hematogenous spread, or extension from breast ducts transected during surgery. Among these, airborne dissemination of *Aspergillus* spores at the time of implantation, with subsequent contamination of the saline and implant surface, is considered the most likely route of infection. The susceptibility of saline-filled implants to microbial contamination has been confirmed in both in vitro and in vivo studies. Notably, *A. fumigatus* has been shown to survive within saline-filled implants for up to six months.

There is increasing evidence that the envelopes of saline-filled breast implants and tissue expanders function as semi-permeable membranes. This has been demonstrated in vitro and corroborated by studies analyzing saline aspirated from implants in patients. Specifically, saline-filled implants have been shown to permit diffusion of oxygen, carbon dioxide, water, electrolytes (including potassium), glucose, proteins, antibiotics, and steroids. The elevated albumin concentration and electrolyte shifts toward serum levels observed in implant fluid suggest that fungal activity may further compromise the silicone shell, increasing its permeability. This aligns with reports associating saline-filled implants with fungal infection, implying that their semi-permeable nature may create a microenvironment conducive to fungal growth.

Across reported cases, a consistent inflammatory response has been documented in the soft tissue surrounding breast implants complicated by fungal infection. Histopathological descriptions often include granulomatous reactions, lymphocytic infiltrates, and chronic capsule remodeling, reflecting a sustained local immune response. One proposed concern is that filamentous fungal biofilms may have a greater capacity for tissue or capsular surface invasion than bacterial or yeast biofilms, although direct implant-specific evidence remains limited. The mechanism of fungal involvement in breast implant capsule infection appears multifactorial, with possible sources including airborne dissemination, contamination during manufacture, hematogenous spread, or extension from breast ducts transected during surgery, as noted in [16]. Once infection occurs, filamentous fungi may form biofilms that could contribute to capsular involvement and related local changes. A comprehensive review of silicone breast implants [6] highlighted that silicone can migrate within the body, raising questions about the permeability of silicone implant shells and their potential interaction with surrounding tissues. One leading hypothesis proposes that chronic, low-grade infections and biofilm formation on or around implants may contribute to persistent inflammation and immune dysregulation [17]. Collectively, these structural and inflammatory changes provide a plausible mechanistic link to clinical manifestations such as capsular contracture, delayed peri-implant swelling, and, in some patients, systemic inflammatory symptoms, although this remains to be confirmed in larger studies.

Infections caused by filamentous fungi, including *Penicillium* spp. and *Aspergillus* spp.—particularly *A. flavus*—have raised concern regarding their potential contribution to inflammatory changes in the peri-implant environment. These organisms are capable of producing mycotoxins and inducing chronic inflammatory responses; however, the extent to which fungal colonisation or mycotoxin exposure contributes to breast-implant-associated inflammation remains uncertain. Laboratory studies have shown that individuals exposed to indoor mould may develop measurable immunoglobulin G or immunoglobulin E antibody responses to specific mycotoxins, suggesting immune sensitisation and the possibility of chronic exposure [18]. Current evidence does not clarify whether such inflammatory effects arise from fungi alone or occur in combination with bacterial or polymicrobial communities, nor whether intact implant membranes allow meaningful interaction with fungal structures. At present, the relationship between filamentous fungal infection, mycotoxin exposure, and peri-implant inflammation is best regarded as an emerging and biologically plausible hypothesis rather than an established mechanism.

Finally, the reason most reports describe these cases as the first documented breast-implant-associated fungal infection is that such infections have only recently begun to be recognized. This is consistent with the observation in [13] that approximately one quarter of breast implant infections yield negative cultures—likely related to the limitations of standard culture media—and that fungal cultures are not routinely performed. In this context, Table 1 summarizes the clinical presentations and intraoperative pathological findings extracted from the reported cases, which may help clinicians identify features suggestive of filamentous fungal involvement. When such features are present, further diagnostic evaluation should include fungal cultures with extended incubation, targeted histopathology using appropriate stains, and molecular assays where available. Tissue or peri-implant fluid should be sampled even when no clear explanation is immediately apparent, as routine diagnostic methods may fail to detect fungi or biofilm-associated organisms. Given the small number of reported cases, these proposed approaches are based on available observations and clinical judgment rather than standardized protocols. Earlier recognition of possible fungal involvement may help guide more targeted investigation and management in selected cases.

## Figures and Tables

**Table 1:** Reported Cases of Filamentous Fungal Breast Implant Infection.

Source	Time From Implantation	Clinical Presentation	Intraoperative/Pathologic Findings	Microbiology and Key Interpretive Points
Kuhn & Homsy (2022) [13]	20 years	Unilateral breast swelling, tenderness, erythema; systemic symptoms (fatigue, cognitive complaints, unusual body odor, and difficulty losing weight); and severe capsular contracture	Purulent drainage, white surface coating, and turbid green fluid with debris	*Propionibacterium acnes* and unspecified *Penicillium* species. The authors note fungal involvement may be under-recognized due to limited routine fungal cultures.
Koan et al. (2022) [14]	5 years	Bilateral swelling, tenderness, and purulent fluid collections	Implants removed prior to culture; histopathology performed	Expands known spectrum to *Scedosporium apiospermum*. Authors emphasize that most implant infections are bacterial (e.g., *S. aureus*), but filamentous fungi should be considered in late-onset complications; culture is essential due to nonspecific histology.
Dinolfo (2013) [15]	Early postoperative period (persistent infection)	Erythema, induration, and purulent drainage unresponsive to antibiotics	Prosthesis completely encased in mold	*Aspergillus fumigatus* cultured. Infection attributed to intraoperative or postoperative contamination; no evidence of manufacturer defects, suggesting an isolated contamination event.
Wright et al. (2006) [16]	18 months	Sudden enlargement and pain over three days	Intact implant surrounded by gelatinous yellow fluid; turbid material within implant lumen	*Aspergillus flavus* cultured from luminal and pericapsular fluid. Histopathology showed lymphocytic infiltrate and granulomas with branching hyphae. Authors discuss multiple contamination routes and highlight semipermeability of saline implants, supporting fungal ingress and survival.

## Data Availability

No new data were created or analyzed in this study. Data sharing is not applicable to this article.
